# Automated segmentation of hepatic vessels and lobules in whole-slide images using U-net models

**DOI:** 10.3389/fbinf.2026.1713736

**Published:** 2026-04-30

**Authors:** Mehul Bafna, Matthias König, Sylvia Saalfeld, Vladimira Moulisova, Vaclav Liska, Uta Dahmen, Mohamed Albadry

**Affiliations:** 1 Experimental Transplantation Surgery, Department of General, Visceral and Vascular Surgery, University Hospital Jena, Jena, Germany; 2 Institute for Biology, Faculty of Life Science, Humboldt-Universität zu Berlin, Berlin, Germany; 3 Institute of Structural Mechanics and Dynamics in Aerospace Engineering, University of Stuttgart, Stuttgart, Germany; 4 Department of Medical Informatics, University Medical Center Schleswig-Holstein, Kiel, Germany; 5 Biomedical Center, Faculty of Medicine in Pilsen, Charles University, Pilsen, Czech Republic; 6 Department of Surgery, Faculty of Medicine in Pilsen, Charles University, Pilsen, Czech Republic; 7 Department of Pathology, Faculty of Veterinary Medicine, Menoufia University, Menoufia, Egypt

**Keywords:** deep learning, hepatic vascular segmentation, image analysis, lobule segmentation, neural networks, machine learning, artificial intelligence

## Abstract

Automated analysis of hepatic vascular structures and lobules within whole-slide histological images is critical for ensuring accurate and timely morphometric evaluations and facilitating advancements in computational liver histology. Nonetheless, the intricate morphology of the tissue, variability in staining techniques, and the requirements for standard high-resolution images present substantial challenges to the precision of segmentation processes. We present a robust deep-learning pipeline using adaptive patch extraction and specialized nnU-Net architectures for segmenting vessels, bile ducts, and lobules in Glutamine Synthetase and Picro-Sirius-Red stained porcine liver sections. Our architecture incorporates a weight-boosted nnU-Net framework with an adaptive, performance-based weight adjustment mechanism to effectively manage class imbalances and improve the detection of smaller vascular structures. The model was trained on four annotated whole-slide images and validated through comprehensive testing on eight additional independent slides. Geometric and intensity-based data transformations enhanced the robustness and generalizability of the segmentation models. Evaluations conducted through five-fold cross-validation, as well as assessments utilizing independent test datasets, resulted in Dice similarity scores: 0.968 for lobules, 0.795 for central veins, 0.895 for hepatic arteries, 0.665 for portal veins, and 0.694 for bile ducts. The developed segmentation pipeline additionally supports comprehensive morphometric analyses of structural parameters, including number and size (diameter, area) of vascular structures, bile ducts, and lobules; for example, the diameter of hepatic arteries ranges between 20–90 µm. These findings underscore the practical relevance of adaptable segmentation frameworks in advancing computational histological analysis of liver tissue.

## 1 Introduction

The liver is a highly vascularized organ responsible for critical physiological functions, including metabolism, detoxification, and immune regulation (Asrani et al., 2019). Key histological features of the liver include vascular components—such as portal veins, central veins, and hepatic arteries—and non-vascular structures like bile ducts, all of which are organized within hepatic lobuli, the fundamental structural and functional units of the liver (Trefts et al., 2017). Prior work has examined structural elements such as hepatic lobules, without focusing on vascular structures (Albadry et al., 2024).

Advances in digital histology and whole-slide imaging (WSI) have enabled automated analysis of liver tissue. However, accurately segmenting both vascular structures and lobular boundaries simultaneously remains challenging. Liver tissue segmentation presents several challenges due to the complex anatomical and histological characteristics of hepatic structures. Low contrast regions, often caused by inconsistent staining and focal tissue artifacts, can obscure the boundaries between vessels and surrounding parenchyma, making the delineation of small arteries and bile ducts particularly difficult (Cullen and Stalker, 2016). Additionally, the wide range of scales among vascular structures introduces further complexity, as a segmentation approach must capture the fine-grained details of small vessels while also accurately identifying larger, branching structures. Performance often varies with size: smaller vessels, being contained within individual image patches, are typically segmented more accurately, whereas larger vessels that span multiple patches are susceptible to fragmentation and misclassification. The morphological similarity between different hepatic components, such as vascular structures and bile ducts, further increases class ambiguity, complicating accurate differentiation and lobular recognition (Invernizzi, 2013). Moreover, effective segmentation is heavily dependent on contextual cues, as structures like portal fields and central veins provide critical spatial references. Due to these difficulties, most of the previous studies focus either on lobule detection or the identification of vascular structures, which limits their applicability.

Most existing vascular segmentation studies targeting the liver focus on radiological modalities, particularly Magnetic Resonance Imaging (MRI) and Computed Tomography (CT). A thorough examination of various computational methods for liver vessel segmentation, encompassing both traditional image processing and modern deep learning techniques, is provided in the review (Ciecholewski and KassjaÅ,ski, 2021). The effectiveness of nnU-Net and its variants for liver segmentation tasks has been demonstrated prior in MRI and CT images, e.g., (Hille et al., 2024; 2023) and occasionally in histological images, e.g., Jirik et al. (2020). Nevertheless, the research gap remains limited for vascular segmentation within histological images.

Therefore, we propose a regularized nnU-Net architecture to segment both hepatic vascular and non-vascular structures as well as lobular boundaries effectively in whole slide images from histological liver sections. Integrating deep learning into the segmentation framework enhances the ability to delineate intricate vascular and non-vascular structures and heterogeneous lobular patterns, thereby improving accuracy across multiple spatial scales.

The porcine liver utilized in the study serves as an ideal experimental model due to the clear delineation of hepatic lobules by collagen-rich septae, facilitating accurate segmentation of vascular structures and offering essential contextual information for defining lobular organization (Meurens et al., 2012). It provides an effective foundation for the future development of segmentation algorithms that are transferable across species and adaptable to diverse histological conditions.

## 2 Materials and methods

### 2.1 Dataset preparation

Liver samples were explanted from Prestice black-pied pigs (weighing 25–33 kg) under general anesthesia and immediately fixed in formalin with no ischemia time. The work with animals was conducted under the law of the Czech Republic, which is in line with the legislation of the European Union. The procedure protocol was approved by the Ministry of Education, Youth and Sport of the Czech Republic (approval no. MSMT-15629/2020-4). Liver tissue samples were obtained from male and female young donors who were sacrificed for other reasons, in compliance with animal regulations and 3R principles (Replacement, Reduction, and Refinement).

Four whole slide images (WSIs) of liver biopsies from four clinically healthy, 3-month-old Prestice Black-Pied pigs (both male and female) were selected for model development. These slides exhibited natural variations in staining intensity due to differences in tissue processing and immunohistochemistry reactions. Eight additional WSIs (with and without artifacts), obtained from three clinically healthy Prestice Black-Pied pigs (both male and female) of the same age and weight range, were used for independent testing. In total, we analyzed tissue from five unique animals (two animals contributed different liver sections to both training and testing sets, ensuring spatial independence). Briefly, liver sections were cut at 3 µm thickness and processed using a standardized immunohistochemistry protocol. Immunostaining was performed using a mouse monoclonal anti-glutamine synthetase (GS) antibody (MAB302; 1:10,000 dilution; Merck, Germany), followed by counterstaining with Picro-Sirius Red (PSR; 1342500250, Morphisto, Germany). Detection was carried out using an avidin-biotin system consisting of a biotinylated goat anti-mouse IgG H&L (ab6788, Abcam, Germany), Streptavidin-HRP (ab64269, Abcam, Germany), and an avidin/biotin blocking kit (ab64212, Abcam, Germany). DAB chromogen (GV825, Dako, Denmark) was used for signal visualization.

GS staining reveals a characteristic perivenous hepatocyte distribution in the liver, aiding in hepatic zonation analysis (Norenberg, 1979). PSR staining was performed as counterstain which produces a distinctive red coloration of collagen fibers under brightfield microscopy (Lattouf et al., 2014). In addition, it provides excellent contrast for vessel wall delineation compared to standard HE staining (Junqueira et al., 1979).

Slides were digitized at a resolution: 227 nm/pixel using a Hamamatsu NanoZoomer scanner (model L11600) with NDP.view2 software. Annotations for both training and test datasets were performed by trained lab personnel using the Hamamatsu NDP.view2 annotation toolbox, following standardized anatomical criteria for structure identification. Below is the list of set anatomical criteria for structure identification.

- Lobules: Polygonal structures (400–1,000 µm diameter) bounded by collagen-rich septa visible as red-stained boundaries (PSR staining)
- Portal Veins: Large vessels (>80 µm) with thin walls, located within portal triads
- Hepatic Arteries: Small to medium vessels (10–90 µm) with thick muscular walls, within portal triads
- Bile Ducts: Small tubular structures (10–30 µm) lined by simple epithelium with visible nuclei, within portal triads
- Central Veins: Thin-walled vessels (30–60 µm) at lobule centers, surrounded by GS-positive hepatocytes (brown staining)

To ensure annotation quality and consistency, completed annotations were randomly reviewed and verified by experienced life scientists (UD, MA), who provided feedback and corrections when necessary. All annotations were completed within a consistent timeframe as part of the dataset preparation phase prior to any model development, ensuring independence from model predictions and eliminating potential annotation bias. Due to the large WSI sizes, training was conducted on smaller patches. The WSI was divided into non-overlapping patches using the OpenSlide module (version 1.2.0) in Python to facilitate training (Goode et al., 2022).

#### 2.1.1 Vessel segmentation dataset

**FIGURE 1:**
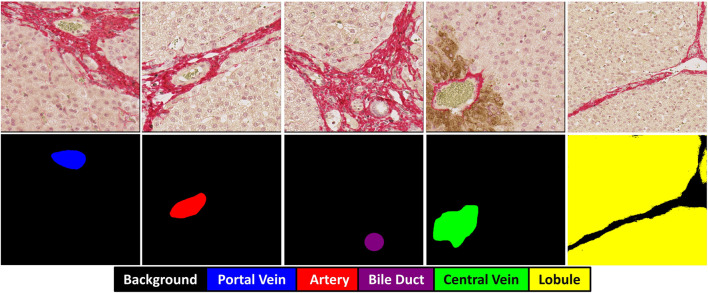
Overview of original image and ground truth segmentation. From top to bottom: Original patch and its corresponding ground truth multi-class mask.

For vessel segmentation, image patches were extracted at a size of 1024 × 1024 pixels to match the input dimension requirements of the nnU-Net architecture. This ensured compatibility with the downsampling and upsampling operations (based on a factor of $2^{n}$ , where n = 5) of the network and helped prevent information loss at patch boundaries (Isensee et al., 2018). All patches were saved in Tagged Image File Format (TIFF) with lossless compression to preserve image quality. The vessel segmentation dataset consisted of 7,272 non-overlapping patches extracted from four WSIs. A random 10% (728 patches) was reserved for testing, while the remaining 6,544 patches (90%) were used for five-fold cross-validation. In each fold, 5,236 patches were used for training and 1,308 for validation with random horizontal/vertical flips, rotations ([30°,60°,90°,120°,150°,180°,and  270°]) , and intensity-based transformations applied for data augmentation. Each image patch was paired with a manually annotated segmentation mask containing five classes: background (black pixel, value 0), portal vein (blue pixel, value 1), central vein (green pixel, value 2), hepatic artery (red pixel, value 3), and bile duct (purple pixel, value 4) (refer to Figure 1). The masks were saved in TIFF format with the same spatial dimensions as the corresponding image patches.

#### 2.1.2 Lobule segmentation dataset

For hepatic lobule detection, a separate dataset of 7500 non-overlapping patches was prepared with a larger dimension of 2048 × 2048 pixels. The increased patch size was specifically chosen as lobules represent larger structural units compared to individual vessels. Each lobule patch was saved in TIFF format with corresponding binary masks, where yellow represents lobule regions as shown in Figure 1. Similar to the hepatic vascular segmentation model, a random 10% split was used to select 750 patches for testing. The remaining 6750 patches (90% of the dataset) were used for five-fold cross-validation. In each iteration, 5400 patches (four folds) were used for training and 1350 patches (one fold) for validation, applying the same geometric data transformations as in the vessel segmentation model.

The class distribution in the dataset for Network 1 was highly imbalanced, with background occupying 89.81% of the total pixels (refer to Table 1). This strong class imbalance was decisive for the choice of loss function and data augmentation strategies.

**TABLE 1:** Overview of annotated structures and their pixel-wise representation in vessel and lobule segmentation networks.

Structure type	Total structures annotated	Pixel proportion (% of total image pixels)
Network 1: Vessel segmentation		
Background	—	89.81%
Portal vein	2991	5.76%
Artery	1713	1.25%
Bile duct	2094	0.75%
Central vein	969	2.42%
Network 2: Lobule detection		
Background	—	26.29%
Lobule	1998	73.71%

#### 2.1.3 Evaluation strategy

To ensure unbiased and comprehensive evaluation of model performance, we employed a rigorous multi-level evaluation strategy for both vessel and lobule segmentation models:

Training and Cross-Validation: Four whole slide images from clinically healthy pigs were used to generate patches for both segmentation tasks. All annotations were performed by trained lab personnel as part of the dataset preparation phase, prior to any model development, ensuring complete independence from model outputs. From the generated patches, 90% were used for model training with 5-fold cross-validation, while the remaining 10% were held out as an independent internal test set, completely excluded from both training and cross-validation processes.

Independent Testing on External Images: Additionally, eight completely unseen whole slide images (with and without artifacts) were used as an independent external test set to evaluate both model generalizability and morphometric measurement consistency. These slides were collected independently and were not part of the original dataset used. This test set assesses model robustness across different animals, tissue samples, and potential variations in tissue processing and staining intensity, while simultaneously enabling comprehensive morphometric analysis to quantify segmentation reliability and measurement accuracy on independent data.

**FIGURE 2:**
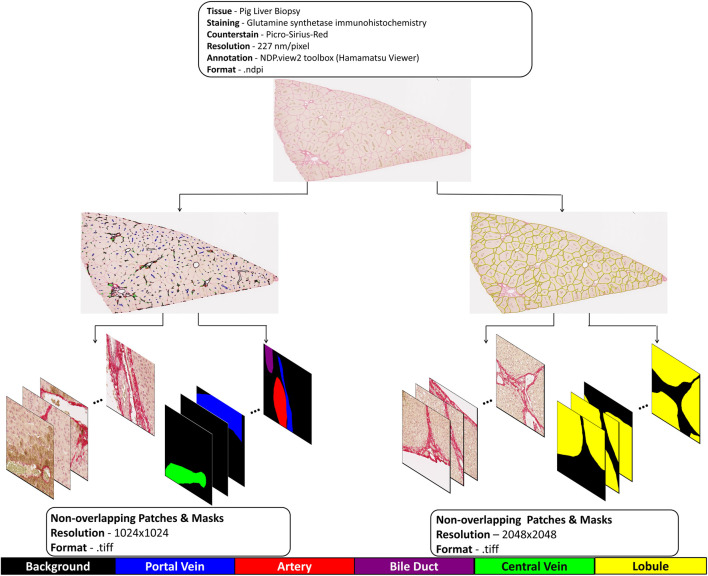
Stepwise breakdown of dataset preparation. Dataset Preparation Workflow: Stage 1 – WSI Generation; Stage 2 – Separate Annotations for Hepatic Vessel Segmentation Training and Lobule Segmentation Training; Stage 3 – Extraction of Annotated Patches and Corresponding Ground Truth Masks from WSIs.

This multi-level strategy ensures comprehensive model evaluation: 5-fold cross-validation provides robust performance estimates during model development, held-out patches from the training WSIs assess internal validity, and the independent external test set evaluates true generalization to different animals and tissue samples. All evaluation sets were kept strictly separate from training data, eliminating potential bias in performance assessment. The complete end-to-end dataset preparation pipeline is illustrated in Figure 2. To accommodate the distinct spatial scales of hepatic structures, different patch sizes were employed: smaller patches (1024 × 1024 pixels) for individual vascular structures and larger patches (2048 × 2048 pixels) for lobular regions.

### 2.2 Network architecture

We propose two state-of-the-art, distinct segmentation models: a weight-boosted nnU-Net architecture for liver vessel segmentation and a standard nnU-Net model for hepatic lobule segmentation. Separate networks for vessel and lobule segmentation were employed to prevent feature confusion and improve specificity, particularly since central veins are located within the hepatic lobule.

#### 2.2.1 Vessel segmentation network

**FIGURE 3:**
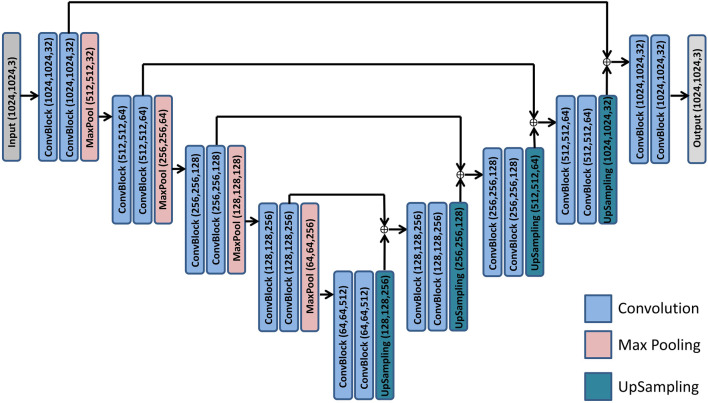
Model architecture used for segmentation tasks. Architecture of the nnU-Net model used in vessel segmentation and lobule detection.

The vessel segmentation network is based on the nnU-Net architecture (Isensee et al., 2018) integrated with an adaptive weight-boosting mechanism to address the strong class imbalance between vessel and background pixels. The model processes input images of size 1024 × 1024 × 3 through an encoder with four blocks, each containing a double convolutional layer (DoubleConv) followed by max-pooling, progressively increasing feature channels (32 →64→128→ 256) and reducing spatial dimensions (refer to Figure 3). The encoder ends with a bottleneck layer (64 × 64 × 512) for feature processing. The decoder mirrors the encoder, with upsampling blocks that sequentially reduce feature channels (256 →128→64→ 32) while restoring spatial details. The weight-boosting mechanism dynamically adjusts the class-wise loss contributions during training, ensuring proper learning despite the underrepresented vessel class.

#### 2.2.2 Lobule segmentation network

The second network focuses on hepatic lobule detection and is built upon the same nnU-Net architectural framework. Unlike the vessel segmentation network, this model does not implement weight-boosting mechanisms as the class distribution is less severely imbalanced. The lobule segmentation network maintains the same encoder-decoder structure with four blocks in each path and identical feature channel progression. Skip connections between corresponding encoder and decoder layers facilitate the preservation of spatial information throughout the network, which is essential for accurately delineating lobular boundaries.

Although both networks leverage the nnU-Net framework, the key difference lies in the optimization strategy, with the vessel segmentation network specifically adjusted to handle the challenges of segmenting fine vascular structures that constitute a small fraction of the overall image area.

### 2.3 Experiments

All experiments were conducted on the Draco HPC cluster (Friedrich Schiller University Jena, Germany) using NVIDIA A100 GPUs with 40 GB memory, managed through the Slurm workload management system. The software environment comprised Python version 3.10, PyTorch version 2.6, and CUDA 11.8.

Training Time: For 5-fold cross-validation with 250 epochs, the vessel segmentation model required approximately 70 h of total training time. In contrast, the lobule segmentation model required more time, with approximately 72 h of training time. Training time represents wall-clock time for complete cross-validation, including all validation evaluations and model checkpointing. Training was accelerated through data parallelism across 2 GPUs and mixed precision training. These hyperparameters were determined through empirical validation, evaluating combinations of batch sizes [4, 8, 16], learning rates [0.0001, 0.0005, 0.001], and optimizers [Adam, SGD with momentum].

Inference Time: For a typical whole-slide image with approximate dimensions of 100,000 × 65,000 pixels, corresponding to 310 ${\text{mm}}^{2}$ (resolution: 0.227 µm/pixel), the complete segmentation pipeline requires approximately 2.5–3 h of computation time on a single NVIDIA A100 GPU. This comprises: vessel segmentation inference (∼30 min), lobule segmentation inference (∼30 min), blending of overlapping patch predictions (∼30–40 min), overlay generation for visualization (∼30–40 min), and final image merging (∼25–30 min). The computational time scales with WSI dimensions and resolution. Both nnU-Net variants were trained with the following hyperparameters.

- Epochs: 250
- Batch size: 8
- Optimizer: Adam’s optimizer with weight decay 0.0002
- Learning rate: 0.0005 (Cosine Annealing)

These hyperparameters were determined through empirical validation, evaluating combinations of batch sizes [4, 8, 16], learning rates [0.0001, 0.0005, 0.001], and optimizers [Adam, SGD with momentum]. Cosine annealing is a learning rate scheduling strategy that smoothly decreases the learning rate following a cosine curve. This gradual decay helps the model converge more effectively by avoiding abrupt changes in the learning rate. The learning rate $\eta_{t}$ at epoch t is given by the formula:

$$\eta_{t}=\eta_{\text{min}}+\frac{1}{2}\left(\eta_{\text{max}}-\eta_{\text{min}}\right)\left(1+\cos\left(\frac{t}{T}\pi \right)\right)$$

where:

-
$\eta_{\text{max}}$ is the initial (maximum) learning rate,
-
$\eta_{\text{min}}$ is the minimum learning rate,
-
T is the total number of epochs or iterations.

To address the class imbalance inherent in the whole-slide images where background pixels substantially outnumber vessel pixels, an adaptive weight-boosting mechanism was applied during the training of the hepatic vessel segmentation model. The algorithm dynamically adjusted class weights every 15 epochs based on class-wise Dice scores: for classes with Dice scores below 0.3, weights were multiplied by a factor of 1.3; for classes with scores between 0.3 and 0.5, weights were multiplied by 1.2; and for classes with scores between 0.5 and 0.7, weights were multiplied by 1.1. This weight adjustment scheme was determined by evaluating different combinations of multiplication factors ([1.1, 1.3, 1.5] and [1.1, 1.2, 1.3]) and Dice score thresholds ([0.3, 0.5, 0.7] and [0.2, 0.4, 0.6]). The selected configuration uses multiplication factors of [1.1, 1.2, 1.3] at thresholds [0.3, 0.5, 0.7], providing optimal balance between convergence speed and segmentation performance across all vessel classes.

**FIGURE 4:**
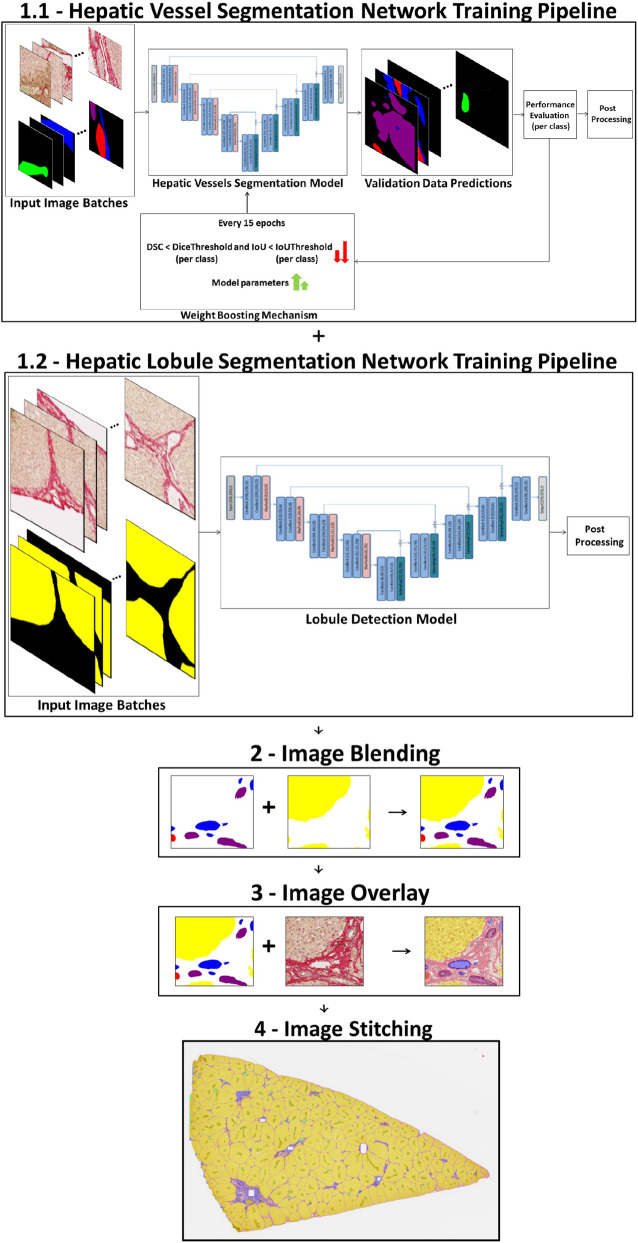
End-to-end pipeline for segmentation and integration. Overview of the end-to-end dataset preparation pipeline for hepatic tissue analysis. The process begins with two independent segmentation models: (1.1) a hepatic vessel segmentation network that identifies vascular structures and (1.2) a hepatic lobule segmentation network that detects lobular regions. Each model is trained separately using annotated input image batches, followed by performance evaluation and post-processing of the respective predictions. In the second stage, the resulting segmentation masks are integrated through a series of steps—image blending (Step 2), image overlay on histological input (Step 3), and image stitching (Step 4)—to generate a comprehensive, standard high-resolution whole-slide representation of hepatic tissue combining vascular and lobular features.

For vessel segmentation, a weighted combination of Dice, focal, and cross-entropy losses was applied with weights of $w_{CE}=0.2$ for cross-entropy, $w_{\mathrm{Dice}}=0.6$ for Dice loss, and $w_{\mathrm{Focal}}=0.2$ for focal loss $\left(w_{CE}\times CE+w_{\mathrm{Dice}}\times Dice+w_{\mathrm{Focal}}\times Focal\right)$ . In contrast, the lobule detection model utilized a simpler weighted binary cross-entropy with Dice loss formulation with $w_{CE}=w_{\mathrm{Dice}}=0.5$ $\left(w_{CE}\times CE+w_{\mathrm{Dice}}\times Dice\right)$ , as the pixel distribution was less imbalanced (refer to Figure 4). Additionally, to handle overfitting, an early stopping mechanism was implemented, terminating training if the validation loss showed no improvement over 20 consecutive epochs. Furthermore, 5-fold cross-validation was employed to ensure robust evaluation and generate reliable final results across the complete dataset.

### 2.4 Metrics

To evaluate the segmentation performance, we utilized the Dice Similarity Coefficient (Dice score), Intersection over Union (IoU), True Positive Rate (TPR), and Average Symmetric Surface Distance (ASSD). These metrics are widely used in medical image analysis to quantify overlap and accuracy between predicted and ground truth segmentations.

#### 2.4.1 Dice similarity coefficient (DSC)

The Dice score measures the overlap between the predicted segmentation P and the ground truth G, and is defined as in Equation 1:

$$\text{DSC}=\frac{2|P\cap G|}{\left|P|+|G\right|}$$ (1)

For a specific class c, the class-wise Dice score is given by Equation 2:

$${\text{DSC}}_{c}=\frac{2|P_{c}\cap G_{c}|}{\left|P_{c}|+|G_{c}\right|}$$ (2)

#### 2.4.2 Intersection over union (IoU)

IoU, also known as the Jaccard index, is defined as the ratio of the intersection over the union of the predicted and ground truth regions as in Equation 3:

$$\text{IoU}=\frac{\left|P\cap G\right|}{\left|P\cup G\right|}$$ (3)

For a specific class c, the class-wise IoU is given by Equation 4:

$${\text{IoU}}_{c}=\frac{\left|P_{c}\cap G_{c}\right|}{\left|P_{c}\cup G_{c}\right|}$$ (4)

#### 2.4.3 True positive rate (TPR)

The True Positive Rate measures the proportion of actual pixels for a specific class that are correctly identified by the model. Given a predicted segmentation P and the ground truth G , the TPR is defined as in Equation 5:

$$\text{TPR}=\frac{TP}{TP+FN}$$ (5)

For a specific class c, the class-wise TPR is given by Equation 6:

$${\text{TPR}}_{c}=\frac{TP_{c}}{TP_{c}+FN_{c}}$$ (6)

where TP denotes the number of true positives (correctly predicted positive pixels) and FN is the number of false negatives (missed positive pixels) for that class.

#### 2.4.4 Average symmetric surface distance (ASSD)

The Average Symmetric Surface Distance quantifies the mean distance between the surface boundaries of the predicted segmentation P and the ground truth G. It is computed as the average of distances from each point on one surface to the closest point on the other surface, considering both directions to ensure symmetry as shown in Equation 7:

$$\text{ASSD}=\frac{1}{2}\left(\frac{1}{\left|S_{P}\right|}\underset{p\in S_{P}}{\sum }d\left(p,S_{G}\right)+\frac{1}{\left|S_{G}\right|}\underset{g\in S_{G}}{\sum }d\left(g,S_{P}\right)\right)$$ (7)

where $S_{P}$ and $S_{G}$ represent the surface boundaries of the predicted and ground truth segmentations respectively, $\left|S_{P}\right|$ and $\left|S_{G}\right|$ denote the number of surface points, and $d\left(p,S_{G}\right)$ represents the minimum Euclidean distance from point p to any point on surface $S_{G}$ . For a specific class c, the class-wise ASSD is given by Equation 8:

$${\text{ASSD}}_{c}=\frac{1}{2}\left(\frac{1}{\left|S_{P_{c}}\right|}\underset{p\in S_{P_{c}}}{\sum }d\left(p,S_{G_{c}}\right)+\frac{1}{\left|S_{G_{c}}\right|}\underset{g\in S_{G_{c}}}{\sum }d\left(g,S_{P_{c}}\right)\right)$$ (8)

The Dice score and IoU provide insights into the degree of region overlap, while TPR highlights the completeness of the segmentation by measuring how well positive regions are detected. ASSD complements these surface metrics by quantifying boundary precision and evaluating the spatial accuracy of lobule contours.

### 2.5 Morphometric analysis

The overall stitched images compiled either from vessel or from lobule segmentation models were postprocessed prior to morphometric analysis. Determination of the surface area of all vessel structures did not require any further morphometric operations. In contrast, the complete lobule image underwent two morphological operations to avoid underestimation of lobule number due to incomplete separation by septae. Dilation was used to widen the septae and erosion to close eventual remaining gaps. While dilation effectively expanded the septal boundaries, the subsequent erosion process, designed to close remaining discontinuities, resulted in a net contraction of the overall lobular area. Surface areas of the resulting objects representing the lobules was calculated by counting the number of pixel for each object. On both images, area size filters with ranges were applied to reduce the number of false positive counts. For lobules, the size range was set from 12500 $\mu m^{2}$ - 1250000 $\mu m^{2}$ . The upper range was selected to exclude falsely connected lobules. For portal veins the size range was set to at least 1000 $\mu m^{2}$ , for hepatic artery and bile duct, at least 125 $\mu m^{2}$ and for central vein, at least 125 $\mu m^{2}$ . In order to not exclude the large vessels we did not set an upper range. After excluding eventually falsely detected small or very large objects such as interconnected lobules, the total number of all structures within the given area size was determined, for lobules using the lobule segmentation model and vessel structures using the vessel segmentation model. Furthermore, we determined both the Feret maximum and minimum diameter. Feret or caliper diameter refers to the distance between two parallel lines tangent to the projected contour.

Major Feret Diameter - The Feret diameter major axis represents the maximum distance between any two points on the boundary of a structure when measured in all possible directions. This measurement captures the longest dimension of the object, providing insight into its overall size and orientation, as defined in Equation 9.

$$F_{\mathrm{major}}=\max\left(\sqrt{{\left(x_{i}-x_{j}\right)}^{2}+{\left(y_{i}-y_{j}\right)}^{2}}\right)$$ (9)

where $\left(x_{i},y_{i}\right)$ and $\left(x_{j},y_{j}\right)$ are all possible pairs of boundary points.

**FIGURE 5:**
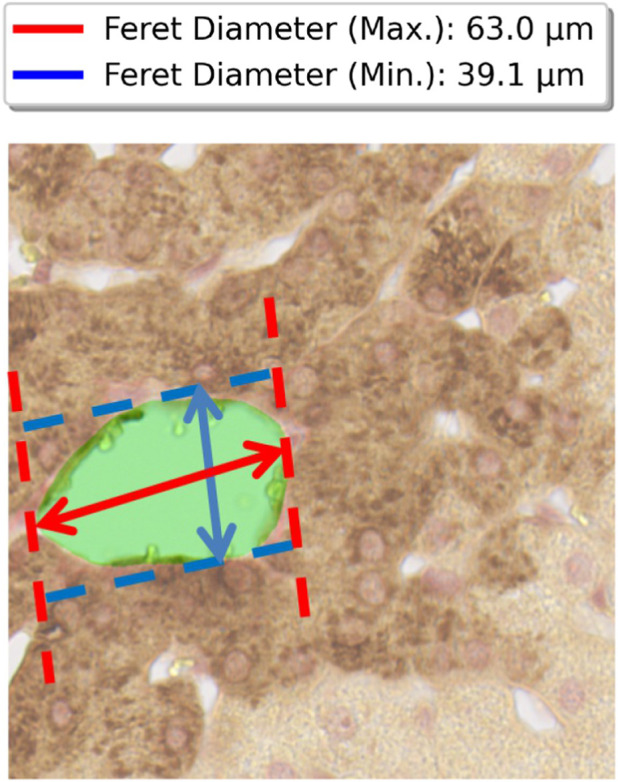
Feret diameter measurements for central vein. Red arrow indicates major axis (maximum boundary distance), blue arrow indicates minor axis (minimum width between parallel lines). Measurements converted to micrometers using 0.227 pixel scaling factor.

Minor Feret Diameter - The Feret diameter minor axis represents the minimum distance between parallel lines that can be drawn to just touch opposite sides of the structure. This measurement captures the narrowest dimension of the object, complementing the major axis to provide a complete picture of the dimensional characteristics for the given structure, as defined in Equation 10.

$$F_{\mathrm{Minor}}=\underset{\theta \in \left[0{}^{\circ},180{}^{\circ}\right]}{\min}\left(\text{width of bounding rectangle at angle }\theta \right)$$ (10)

where θ ranges from 0° to 180°, and the width is measured perpendicular to the longest side at each angle. Below is an illustration of the feret diameters for a segmented central vein in Figure 5.

Aspect ratio was calculated by dividing minimum and maximum Feret diameter.

### 2.6 Code and data availability

To support reproducibility for further research, we have made all relevant resources publicly available. The complete implementation—including deep learning models, data preprocessing scripts, and evaluation tools is hosted on our GitHub repository: https://github.com/MehulBafna/Image-Segmentation-Paper. The repository is organized to reflect each stage of the pipeline and is released under an open-source license.

Our annotated dataset, which includes whole slide images, corresponding image patches, and multiclass segmentation masks for hepatic vessels and lobules, is available through Zenodo: https://doi.org/10.5281/zenodo.19467734.

## 3 Results

### 3.1 Performance metrics

Model performance was evaluated using 5-fold cross-validation (see Table 2) as well as results from the internal test data (see Table 3). The results presented in Table 2 indicate the mean over all the image patches per fold and subsequently over all five cross-validation folds. Both weight-boosted and standard nnU-Net architectures were evaluated to assess the contribution of the adaptive weight-boosting mechanism.

**TABLE 2:** Performance Metrics (Dice vs. IoU vs. TPR) with 5-fold cross validation.

Model	Metric	Portal vein	Artery	Bile duct	Central vein	Lobule
Weight-boosted vessel segmentation nnU-Net	Dice	0.751±0.029	0.805±0.024	0.800±0.049	0.800±0.022	–
	IoU	0.602±0.033	0.674±0.030	0.667±0.059	0.667±0.028	–
	TPR	0.809±0.028	0.834±0.031	0.717±0.016	0.780±0.048	–
Vessel segmentation standard nnU-Net	Dice	0.611±0.032	0.666±0.047	0.623±0.042	0.619±0.055	–
	IoU	0.440±0.033	0.500±0.053	0.452±0.045	0.448±0.058	–
	TPR	0.688±0.043	0.696±0.031	0.683±0.039	0.669±0.048	–
Weight-boosted lobule segmentation nnU-Net	Dice	–	–	–	–	0.959±0.011
	IoU	–	–	–	–	0.921±0.020
	TPR	–	–	–	–	0.944±0.006
Lobule segmentation standard nnU-Net	Dice	–	–	–	–	0.916±0.023
	IoU	–	–	–	–	0.845±0.039
	TPR	–	–	–	–	0.923±0.029

**TABLE 3:** Performance Metrics (Dice vs. IoU vs. TPR) on the internal test data.

Model	Metric	Portal vein	Artery	Bile duct	Central vein	Lobule
Weight-boosted vessel segmentation nnU-Net	Dice	0.665	0.895	0.694	0.795	–
	IoU	0.574	0.814	0.611	0.726	–
	TPR	0.751	0.922	0.744	0.825	–
Vessel segmentation standard nnU-Net	Dice	0.609	0.721	0.593	0.732	–
	IoU	0.448	0.641	0.507	0.577	–
	TPR	0.712	0.823	0.699	0.759	–
Weight-boosted lobule segmentation nnU-Net	Dice	–	–	–	–	0.968
	IoU	–	–	–	–	0.955
	TPR	–	–	–	–	0.977
Lobule segmentation standard nnU-Net	Dice	–	–	–	–	0.873
	IoU	–	–	–	–	0.774
	TPR	–	–	–	–	0.883

The weight-boosted nnU-Net achieved mean Dice scores of 0.75–0.80 for vessel segmentation and 0.959 for lobule segmentation, substantially performing better than the standard nnU-Net (0.61-0.67 for vessels, 0.916 for lobules). The performance was elevated by approximately 13% for vessels and 4.3% for lobules in comparison to the standard nnU-Net. Detailed cross-validation metrics are summarized in Table 2.

**FIGURE 6:**
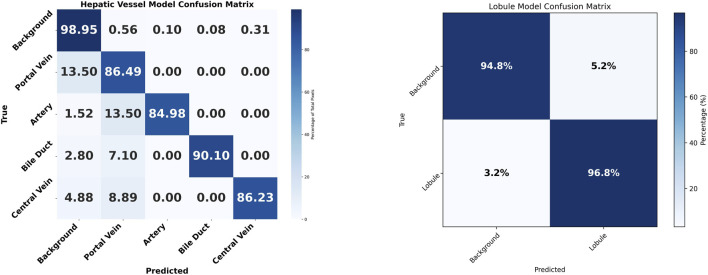
Classification accuracy visualization for both models. Confusion matrices for hepatic vessel and lobule segmentation models. The hepatic vessel model (left) shows classification performance across five classes: Background, Portal Vein, Artery, Bile Duct, and Central Vein. The lobule model (right) demonstrates binary classification performance between Background and Lobule structures. Values represent percentages of true class predictions, with darker blue indicating higher accuracy.

On the internal test dataset, the weight-boosted nnU-Net achieved mean Dice scores of 0.67–0.90 for vessels and 0.968 for lobules, consistently outperforming the standard nnU-Net (0.61–0.73 for vessels, 0.873 for lobules) by 5–12 percentage points. These results on independent internal test data confirm the robustness and generalizability of the weight-boosting mechanism. The detailed results are provided in Table 3, and exemplary segmentations are provided in Supplementary Figure S8. The confusion matrix in Figure 6 illustrates the performance of both models across all classes, emphasizing their classification accuracy and error patterns.

Using the hepatic vessel model, the background is classified with very high accuracy, showing strong model performance in distinguishing the background from the targeted histological structures. Central Veins also have high accuracy (86.2%), with minimal confusion of (4.9%) and (8.9%), primarily with background and portal vein, respectively. Portal Veins have an accuracy of (86.49%), with notable confusion (13.50%) with background, indicating overlap or similarity in features. The arteries are well-identified (84.98%), though 13.50% are misclassified as the portal vein and a small portion as background. The bile ducts have an accuracy of 90.10%, with a substantial portion (7.1%) misclassified as portal vein, suggesting that these two structures are particularly challenging for the model to differentiate.

For the lobule segmentation model, the background achieves excellent classification accuracy (94.8%), though with some confusion (5.2%) with lobule structures. The lobule class demonstrates strong performance (96.8%) with minimal misclassification (3.2%) as background, indicating the model effectively distinguishes lobular architecture from surrounding tissue.

Table 4 provides Average Symmetric Surface Distance (ASSD) in micrometers for the five hepatic structures. Portal veins showed the highest segmentation error (16.96 µm average) with the greatest variability, while central veins achieved the lowest error (2.89 µm) with the most consistent performance. Arteries (5.03 µm), bile ducts (5.99 µm), and lobules (6.03 µm) demonstrated intermediate accuracy levels with ASSD values ranging from 5.03 to 6.03 µm.

**TABLE 4:** Average Symmetric Surface Distance (ASSD) statistics for anatomical structures. Distances are reported in µm, converted from pixels using the original slide resolution of 227 nm/pixel.

Structure	Avg. ASSD	Std. Dev	Median	Min	Max
Portal vein	16.96μm	28.53μm	7.56μm	0.47μm	192.88μm
Artery	5.03μm	4.83μm	2.23μm	0.32μm	18.54μm
Central vein	2.89μm	1.44μm	2.62μm	0.74μm	6.94μm
Bile duct	5.99μm	15.71μm	1.86μm	0.51μm	48.45μm
Lobule	6.03μm	7.30μm	3.72μm	0.96μm	48.48μm

To assess the biological relevance of these ASSD measurements, we calculated relative segmentation errors by comparing boundary errors to typical structure dimensions. Lobules demonstrate remarkably high accuracy with approximately 0.6% relative error (ASSD: 6.03 µm relative to approximately 1,000 µm diameter). Central veins also show excellent accuracy with 6% relative error (ASSD: 2.89 µm relative to 50 µm diameter). Arteries exhibit moderate accuracy with 16% relative error (ASSD: 5.03 µm relative to 30 µm diameter), while portal veins show approximately 17% relative error (ASSD: 16.96 µm relative to approximately 100 µm diameter). These accuracy levels are consistent for morphometric analysis, including vessel counting, approximate diameter quantification, and vessel classification.

### 3.2 Robustness

To assess the robustness of the algorithm towards technical issues possibly occurring in routine histological preparations, we applied the model to eight additional independent WSIs from three different pigs with varying technical quality, including three WSIs with artifacts (with uneven staining, tears, and tissue folds).

**FIGURE 7:**
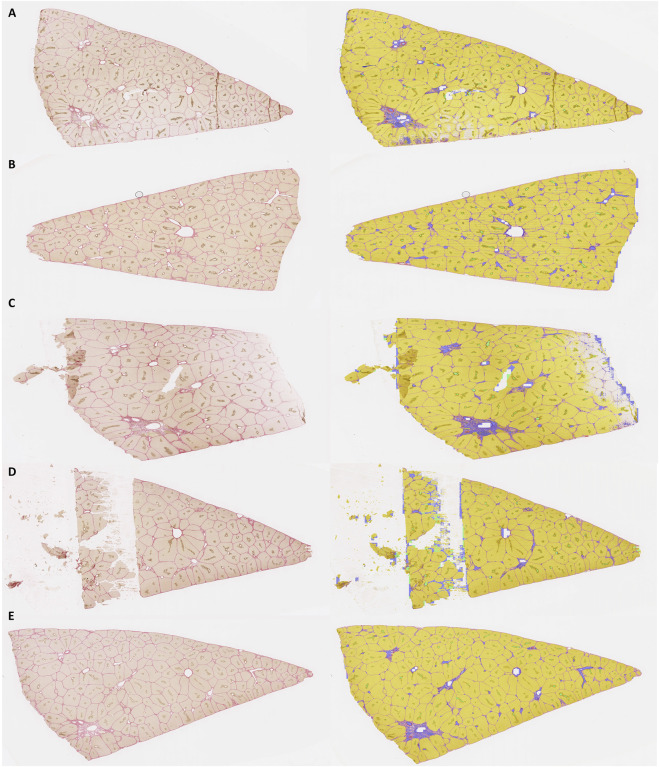
Robustness of segmentation under tissue quality variations. Segmentation robustness across five representative external validation WSIs with varying technical quality. Images show samples with different quality conditions: one with tissue folds (7A), two good-quality samples (7B and 7E), and two with uneven staining and tears as artifacts (7C and 7D). All five structure types (lobules, portal veins, arteries, bile ducts, central veins) are successfully detected across varying quality conditions, with quantitative impact analysis provided in Table 5. Complete overlayed segmented WSIs for all eight images are available on Zenodo https://doi.org/10.5281/zenodo.19467734.

Representative segmentation overlays for five of these WSIs are shown in Figure 7 (two good-quality images, three with uneven staining, tears, and tissue folds). Complete overlayed segmented WSIs for all eight images are available on Zenodo https://doi.org/10.5281/zenodo.19467734. Visual inspection confirms successful segmentation across all eight images despite variations in tissue quality. Segmentation quality was maintained for the majority of tissue area, with localized challenges limited to regions with severe artifacts such as tears or extreme staining irregularities. Quantitative analysis of morphometric consistency across these eight images is presented in the following subsection.

### 3.3 Morphometric analysis

**FIGURE 8:**
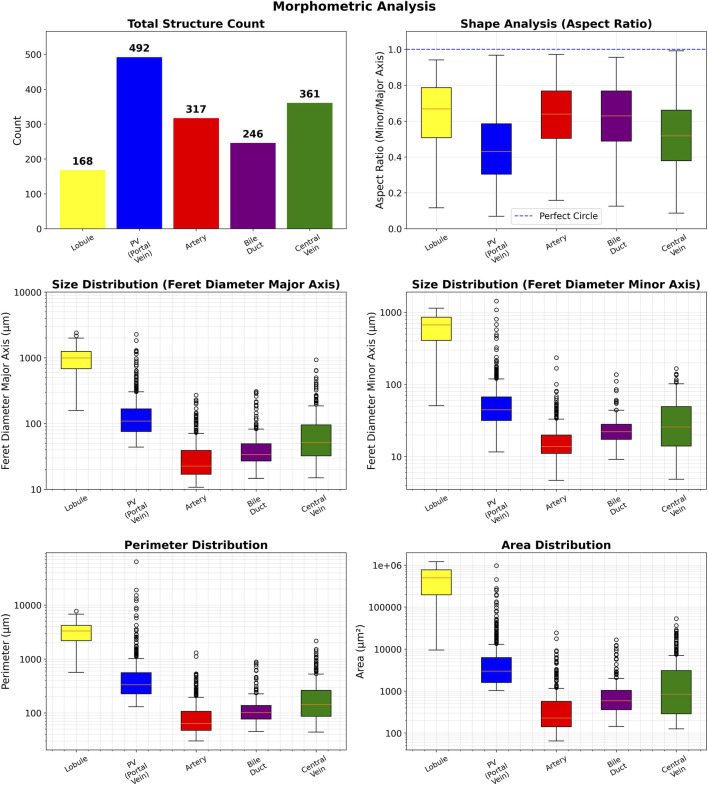
Quantitative structural and spatial analysis of hepatic features. Quantitative parameters derived from histological image analysis include total structure count, shape aspect ratio (minor/major axis), feret diameters (major and minor), perimeter, and area. Aspect ratio analysis indicates deviation from circularity, especially in vessels and bile ducts. Logarithmic scaling was applied to perimeter and area plots for clarity. In the box plots, boxes represent quartiles Q1 and Q3. The upper whisker and lower whisker extend to the last data point less than Q3 + 1.5 ⋅ IQR and the first data point greater than Q1 − 1.5 ⋅ IQR, respectively. IQR denotes interquartile range (Q3−Q1).

To demonstrate both the morphometric capabilities and the robustness of the segmentation framework, comprehensive quantitative analysis was performed on eight independent external WSIs from four different pigs. These unannotated WSIs enabled assessment of the models’ practical utility for large-scale automated morphometric analysis across different tissue samples and varying technical quality conditions. Figure 8 illustrates the results for the morphometric analysis of the liver section depicted in Figure 7E. Complete morphometric analyses for the remaining seven images are provided in Supplementary Figures S1–S7, with quantitative comparisons across all eight images summarized in Table 5.

**TABLE 5:** Quantitative morphometric analysis across eight external validation WSIs grouped by tissue quality. Structure counts, normalized densities, and size measurements with statistical comparisons (two-sample t-test) between artifact-free and artifact-affected groups.

WSI name/Quality	Area	Lobules	Portal	Arteries	Bile	Central
	(mm^2^)		Veins		Ducts	Veins
Structure counts - Artifact-Free WSIs						
J-21-157_46 (Supplementary Figure S2)	200	216	571	144	123	412
J-21-157_28 (Supplementary Figure S3)	180	201	435	260	393	334
J-21-153_2 (Supplementary Figure S6)	130	218	412	264	141	480
J-21-157_8 (Figure 8)	190	168	492	317	246	361
Structure counts - Artifact-Affected WSIs						
J-21-154_2 †,# (Supplementary Figure S1)	125	182	579	397	120	466
J-21-157_4^*^ ^,^ ^#^ (Supplementary Figure S4)	190	170	486	169	296	468
J-21-157_24^†^ ^,^ ^*^ ^,^ ^#^ (Supplementary Figure S5)	138	127	341	224	314	271
J-21-157_20^†^ ^,^ ^*^ ^,^ ^#^ (Supplementary Figure S7)	100	140	266	77	120	311
Normalized density (per mm^2^) - Artifact-Free WSIs						
J-21-157_46	–	1.08	2.85	0.72	0.61	2.06
J-21-157_28	–	1.12	2.42	1.44	2.18	1.86
J-21-153_2	–	1.68	3.17	2.03	1.08	3.69
J-21-157_8	–	0.88	2.59	1.67	1.29	1.90
Mean ± SD	–	1.19±0.34	2.76±0.33	1.47±0.55	1.29±0.66	2.38±0.88
Normalized density (per mm^2^) - Artifact-Affected WSIs						
J-21-154_2^†^ ^,^ ^#^	–	1.46	4.63	3.18	0.96	3.73
J-21-157_4^*^ ^,^ ^#^	–	0.89	2.56	0.89	1.56	2.46
J-21-157_24^†^ ^,^ ^*^ ^,^ ^#^	–	0.92	2.47	1.62	2.28	1.96
J-21-157_20^†^ ^,^ ^*^ ^,^ ^#^	–	1.40	2.66	0.77	1.20	3.11
Mean ± SD	–	1.17±0.30	3.08±1.04	1.61±1.11	1.50±0.57	2.82±0.77
Statistical comparison - Normalized density (AF vs. AA)						
p-value (t-test)	–	0.93	0.57	0.82	0.65	0.48
Major feret diameter (µm) - Artifact-Free WSIs						
Mean ± SD	–	956±85	145±80	28±9	32±4	70±12
Major feret diameter (µm) - Artifact-Affected WSIs						
Mean ± SD	–	733±212	179±78	26±8	34±9	61±14
Statistical comparison - Major feret diameter (AF vs. AA)						
p-value (t-test)	–	0.10	0.56	0.68	0.70	0.36

^†^ Artifacts: Tears.

^*^ Artifacts: Tissue folds.

^#^ Artifacts: Uneven staining.

All p-values >0.05 indicate no significant differences in mean measurements. AF = Artifact-Free, AA = Artifact-Affected.

Morphometric analysis focused on total and relative structure count as well as roundness and size for individual structures. For the representative image shown in Figure 8, portal veins comprised the largest group (n = 492, 31.06%), followed by central veins (n = 361, 22.8%), arteries (n = 317, 20.0%), bile ducts (n = 246, 15.5%), and lobules (n = 168, 10.6%). Shape analysis showed lobules being more circular compared to portal veins, as indicated by higher aspect ratios (median: 0.7 vs. 0.4). Size measurements showed that lobules were substantially larger (median major Feret diameter ≈1,000 μm; area ≈400,000 µ ${\text{m}}^{2}$ ) than vascular structures, with portal veins (median major Feret diameter ≈100 µm) exceeding arteries, bile ducts, and central veins (15–50 µm range).

These quantitative findings illustrate the structural organization of hepatic tissue, spanning multiple scales from large lobules that define tissue architecture to fine vascular structures for microcirculation.

To quantify artifact impact on segmentation performance, we analyzed eight independent WSIs (1,253 mm^2^ total) categorized as artifact-free (n = 4) or artifact-affected (n = 4) containing tears, folds, and/or uneven staining (Table 5). The pipeline detected 11,712 structures across all sections. Statistical comparison revealed no significant differences in normalized structure densities (p = 0.48–0.93, two-sample t-test) or mean major Feret diameters (p = 0.10–0.70) between groups. However, artifact-affected sections showed 2.0–3.2 fold higher variability (standard deviations) for portal veins and arteries, indicating that artifacts primarily affect detection consistency rather than morphometric measurements of correctly identified structures. Supplementary Figures 9–11 illustrate model performance in artifact-affected regions, including small tears/tissue loss, folds, and unevenly stained areas respectively.

## 4 Discussion

Comprehensive morphometric analysis of hepatic morphology calls for the identification and quantification of all histological structures. This study presents a deep-learning framework for segmenting key liver structures, including portal veins, hepatic arteries, bile ducts, central veins, and lobules, in whole slide scans of porcine liver tissue. Our approach did achieve strong segmentation performance independent of size across different types of histological structures within the liver.

### 4.1 Technical comparison with existing approaches

Current hepatic tissue segmentation approaches predominantly target isolated anatomical components, such as bile ducts or portal tracts, limiting their applicability in comprehensive liver tissue analysis (see Table 6).

**TABLE 6:** Technical comparison of existing approaches for vessel segmentation.

Study	Approach	Origin	Tissue condition	Staining	Detected structures	Performance metrics
Su et al. (2022)	Multi-scale attention CNN	Human liver biopsies	Fibrotic (Chronic Hepatitis)	Masson stained	Bile duct	DSC: 0.84IoU: 0.73TPR: 0.86
Yu et al. (2022)	MUSA-UNet	Human transplantation biopsy	Transplant biopsy	HE, masson Trichrome	Portal field	DSC: 0.89IoU: 0.79TPR: 0.85
Budelmann et al. (2022)	Cascade-R-CNN	Mouse liver	Healthy, Resected, steatotic	HE, GS	Lobule, portal field, central vein	DSC: 0.79–0.81IoU: 0.66–0.68TPR: 0.81–0.84
Rong et al. (2023)	MobileNet-v2	Mouse liver	Healthy	GS and zonated markers	Central vein, portal vein	DSC: 0.81–0.89IoU: 0.89–0.94
Bafna et al. (2026)	Weight boosted nnU-Net	Pig liver	Healthy	GS-PSR	Portal vein, central vein, artery, bile duct, lobule	DSC: 0.75–0.96IoU: 0.60–0.92TPR: 0.71–0.94

Four different strategies using deep learning networks for vessel segmentation in WSIs of liver histology were recently reported: Multi-scale attention convolutional network built on a ResNet-101 backbone with the atrous spatial pyramid pooling (ASPP) (Su et al., 2022). This technology builds on dual-magnification input processing with attention mechanisms to enhance feature maps from high-magnification images using low-magnification contextual information. The advantage is the integration of multi-scale spatial information via decoder architecture. However, high computational resource requirements and limited generalization to other vascular and lobular structures represent limitations. Similar to the attention mechanism-based approach in (Su et al., 2022; Yu et al., 2022) proposed the Multiple Up-sampling and Spatial Attention guided U-Net model (MUSA-UNet), which the specifically adapted for portal tract region identification and quantification in transplant biopsy whole-slide images. This modified U-Net architecture incorporates multi-scale attention modules to effectively capture the complex morphological variations present in transplant biopsies. This strategy enhances feature representation and segmentation accuracy, but requires high computational resources due to the integration of attention mechanisms.

The other two approaches are rather different from these encoder-decoder strategies. Budelmann et al. (2022) employed Cascade R-CNN with ResNeXt101 backbone for simultaneous detection of portal fields and central veins using a tile-based approach. Unlike attention-based segmentation networks that enhance feature representation via spatial weighting across scales, the Cascade R-CNN performs structured, multi-stage object detection by the bounding box approach without relying on learned attention maps. Rong et al. (2023) utilized MobileNet-v2 for lightweight vessel detection in healthy tissue. Both approaches rely on object detection paradigms rather than semantic segmentation frameworks. In addition, the approach differs from (Budelmann et al., 2022) as it employs tissue positioning system (TPS), a lightweight semantic segmentation paradigm to generate continuous zonal masks focusing on positional mapping rather than discrete detection.

The comparative performance metrics presented in Table 6 are sourced from the respective published studies and represent results obtained on different datasets, tissue types, and imaging protocols. These comparisons serve to contextualize our segmentation performance within the broader landscape of histological image segmentation rather than providing direct model-to-model evaluation. Cross-study comparisons of published results must be interpreted with appropriate caution, as differences in tissue characteristics, staining protocols, image quality, and annotation granularity can substantially influence segmentation performance.

For lobular segmentation, methodological approaches have evolved from basic geometric principles to sophisticated multi-modal integration (see Table 7). Schwen et al. (2016) utilised a distance-based approach, employing computational geometry to define lobular boundaries through proximity measurements to vascular structures in steatotic mouse liver tissue. Building upon this framework (Laue et al., 2024), introduced distance-based joint zonated quantification. This method combines the geometric principles of distance-based approaches with multi-stain integration capabilities, achieving enhanced zonation mapping accuracy. However, it comes at the cost of substantially increased computational complexity compared to single-parameter approaches.

**TABLE 7:** Technical comparison of existing approaches for lobular segmentation.

Study	Approach	Origin	Tissue condition	Staining	Detected structures	Morphometric metrics
Schwen et al. (2016)	Distance based zonated quantification	Mouse liver	Steatotic	HE, GS	Lobule, zonated quantification	Perimeter: N/A area: 0.13 - 0.35 ${\text{mm}}^{2}$
Peleman et al. (2023)	Voronoi-guided liver lobule mapping	Human liver, mouse liver	Healthy, steatotic	HE, GS	Lobule, lobular zones	Perimeter: N/A area: N/A
Albadry et al. (2024)	Portality based lobular segmentation	Mouse, rat, pig, human liver	Healthy	HE, GS, CYP	Lobule, hepatic zonation	Perimeter^#^: 1.8–3.2 mm Area^#^: 0.3–0.9 ${\text{mm}}^{2}$
Laue et al. (2024)	Distance based joint zonated quantification	Mouse liver	Healthy, steatotic	HE, GS, CYP	Lobule, joint zonated quantification	Perimeter: N/A area: 0.13 - 0.35 ${\text{mm}}^{2}$
Budelmann et al. (2022)	Cascade-R-CNN	Mouse liver	Healthy, Resected, steatotic	HE, GS	Lobule, portal field, central vein	Perimeter: 1.63–1.88 mm area: 0.21 - 0.28 ${\text{mm}}^{2}$
Bafna et al. (2026)	Weight boosted nnU-Net	Pig liver	Healthy	GS-PSR	Portal vein, central vein, artery, bile duct, lobule	Perimeter: 2.2–4.2 mm area: 0.2 - 0.5 ${\text{mm}}^{2}$

^#^ Values correspond to pig liver samples.

Peleman et al. (2023) advanced the methodology by implementing Voronoi-guided mapping strategies. These improved upon simple distance calculations by using tessellation algorithms to create more physiologically accurate lobular zone partitions across both human and mouse samples under healthy and steatotic conditions.

Similar to Table 6, the morphometric measurements presented in Table 7 are sourced from the respective published studies and represent results obtained on different species, tissue conditions, and staining protocols. These comparisons provide contextual benchmarking for our lobular segmentation results, though direct numerical comparisons across different datasets should be interpreted cautiously due to inherent biological and technical variability.

More recent developments have shifted toward comprehensive multi-species applicability. Portality-based segmentation extends beyond the species-limited approaches of earlier methods to encompass mouse, rat, pig, and human tissues (Albadry et al., 2024). This approach further differentiates itself by integrating multiple staining protocols (HE, GS, and CYP) for enhanced lobular analysis. In contrast, previous methods relied predominantly on single-stain approaches. Budelmann et al. (2022) estimated lobular dimensions by utilizing the identified positions of the PF and CV, as mentioned in Table 6 describing their vessel segmentation framework.

In contrast, the present work proposes an integrated multi-class vessel segmentation framework paired with lobule segmentation. The methodology utilizes GS and PSR staining on porcine liver sections to improve the contrast of key anatomical features, such as collagen-dense vessel walls and lobular boundaries. In contrast to other studies mainly focusing on one or few selected structures, this framework simultaneously segments five hepatic components—lobules, portal veins, central veins, hepatic arteries, and bile ducts—within a single pipeline. Our model was developed using four annotated whole-slide images from different animals (both male and female). Although this represents a limited sample in terms of donor diversity, the total number of annotated structures was sufficient to capture the expected morphological variability within healthy young porcine liver tissue. The weight-boosting mechanism specifically targets class imbalance challenges inherent in the hepatic vessel detection with metrics comparable to single-structure methods while maintaining multi-target detection capabilities. Our comprehensive approach represents a substantial advancement in automated hepatic histological analysis, unified for complex multi-structure segmentation tasks.

The proposed weight boosting nnU-Net mechanism demonstrates substantial advancements in terms of vessel segmentation, but also has several biological and technical constraints limiting its current applicability and generalizability. The model was developed using WSI of GS-PSR stained sections from healthy porcine livers which represents a narrow scope of application. Four critical parameters define the operational boundaries of our segmentation framework: species specificity, staining protocol dependency, tissue condition requirements, and section quality standards. Each parameter presents unique challenges that must be addressed to achieve strong performance across diverse scenarios. The selection of nnU-Net as our foundational architecture was guided by its automated configuration pipeline and built-in mechanisms for handling multi-scale structures. This strategy aligned well with our task requirements for the identification of small structures like bile ducts (10–20 µm) up to large structures, such as the lobules (approximately 1,000 µm).

However, we acknowledge that alternative architectures represent viable options with different trade-offs. Transformer-based models such as Swin-UNet (Cao et al., 2022) and UNETR (Hatamizadeh et al., 2022) excel at capturing long-range spatial dependencies through self-attention mechanisms, potentially improving the segmentation of structures with complex spatial relationships. Hybrid CNN-Transformer approaches, such as TransUNet (Chen et al., 2021), combine the local feature-extraction capabilities of CNNs with the global context modeling of transformers. Graph neural networks (Shit et al., 2021; Wolterink et al., 2019) offer specialized architectures for modeling vascular connectivity and topology, which could be particularly relevant for vessel segmentation tasks. Each approach presents distinct trade-offs in terms of data efficiency, computational requirements, and interpretability. Nevertheless, the convolution-based nnU-Net architecture demonstrates superior data efficiency compared to transformer-based segmentation models, particularly relevant given our moderately-sized training dataset, as its built-in spatial inductive biases are well-suited for dense biomedical image segmentation.

An important consideration of our approach is the transferability across species, particularly to rodent and human liver tissue. The porcine liver presents distinct anatomical advantages for algorithm development: hepatic lobules are clearly delineated by collagen-rich septa, providing unambiguous ground truth boundaries for training and validation. In contrast, rodent and human liver tissue lacks visible lobular septa, with lobules merging seamlessly into the parenchyma without clear anatomical demarcation. This fundamental morphological difference presents a substantial challenge for the direct transfer of lobule segmentation models from porcine to rodent and human tissues.

However, several aspects of our methodology remain highly relevant for rodent and human liver analysis. First, the vascular structure segmentation of portal veins, hepatic arteries, central veins, and bile ducts targets morphologically similar features across species and is expected to transfer more readily using domain adaptation techniques or transfer learning approaches. Second, the adaptive weight-boosting mechanism and multi-structure segmentation framework represent methodological contributions applicable across species. Third, in rodent and human liver tissue, the detected vascular landmarks (particularly central veins and portal triads) can serve as inputs for computational approaches to lobular geometry. Here, Voronoi tessellation (Peleman et al., 2023) or distance-based zonation methods are of importance (Schwen et al., 2016; Laue et al., 2024). Both of them define lobular regions through mathematical relationships rather than direct boundary detection. Finally, the expression pattern of additional tissue markers, such as E-cadherin, can provide complementary information for defining, e.g., periportal zones in both rodent and human tissue (Hempel et al., 2015).

### 4.2 Future directions

Taken together, our pipeline enables quantitative vascular architecture assessment and serves as a foundation extending the applicability of the algorithm to other conditions. As a next step, we aim to perform quantitative morphometric analysis across species (mouse, rat, human) and across different stainings, focusing on both tissue morphology and marker protein expression. Further plans include the extension to the total quantification of liver pathologies (hepatic steatosis, fibrosis/cirrhosis, necrosis, inflammation, regeneration), but also the inclusion of zonated quantification of these morphologic changes and, respectively, zonated expression of marker proteins. Application to diseased livers presents substantial challenges, as pathological conditions introduce morphological alterations, including architectural distortion, altered tissue texture, and disrupted vascular organization. Fine-tuning strategies to address these domain shifts could include transfer learning from the healthy tissue model, domain adaptation techniques to bridge the distribution gap, and multi-task learning frameworks that simultaneously segment structures and detect pathological features. Additionally, improving resistance to histological artifacts (tears, folds, uneven staining) through artifact-aware training and preprocessing strategies will be essential for maintaining quantification consistency across varying tissue preparation quality.

To achieve these goals, the key prerequisite on the biological side is the generation of comprehensive annotated datasets across different species, stainings, technical quality, and liver pathologies. Here, datasets covering the whole range of disease severity grades (e.g., fibrosis, steatosis) are needed to enable quantitative assessment with translational potential for disease monitoring. The following four challenges must be addressed. The generalization to multiple species requires enhancing lobular structure identification in species lacking lobular septa. The incorporation of diverse staining methods calls for the implementation of stain normalization, followed by training on differentially stained sections. The applicability to images from sections of suboptimal quality depends on the design of preprocessing pipelines and architectures resilient to artifacts and sectioning flaws. The inclusion of different tissue conditions is the biggest challenge. It requires the annotation of comprehensive datasets representing different severities of one or multiple, even combined disease states.

On the image analysis side, the key steps include staining normalization techniques and domain adaptation methods. Stain normalization refers to the computational adjustment of histological images to reduce color variability introduced by differences in staining protocols or scanners, ensuring consistent feature representation across samples. In this context, domain adaptation addresses cross-species variability by enabling models trained on tissue morphology of one species to generalize effectively to others, such as transferring between human and porcine liver samples. Given the lack of septa in human liver tissue, applying this lobule segmentation approach might be challenging.

## 5 Conclusion

Automated segmentation of hepatic vessels and lobular structures in whole-slide histological images is a fundamental task in computational liver histology, enabling detailed anatomical analysis and supporting downstream image-based quantification. The primary challenge is to correctly identify complex vascular structures (portal veins, central veins, arteries, bile ducts, and lobules) with varying diameters throughout the liver parenchyma.

We implemented two nnU-Net variants enhanced with a weight-boosting mechanism to segment and classify specific hepatic vessel types in whole-slide porcine liver images. Our model is achieving comparable accuracy in specific vessel type classification as reported for general vessel segmentation, while allowing structure-wise morphometric analysis. Nevertheless, the applicability is currently restricted to WSI from GS-PSR-stained high-quality sections from healthy porcine livers.

A coordinated effort to address this main limitation and to extend applicability would substantially advance this field, potentially leading to a robust approach that adapts and transfers efficiently across species, stainings, and pathologies.

## Data Availability

The datasets presented in this study can be found in online repositories. The names of the repository/repositories and accession number(s) can be found below: https://doi.org/10.5281/zenodo.19467734.
