# Supplementary figures and images for: Automated segmentation of hepatic vessels and lobules in whole-slide images using U-net models
Source: Front Bioinform. 2026 Apr 30;6:1713736. doi: 10.3389/fbinf.2026.1713736 (PMC13171784; doi:10.3389/fbinf.2026.1713736)

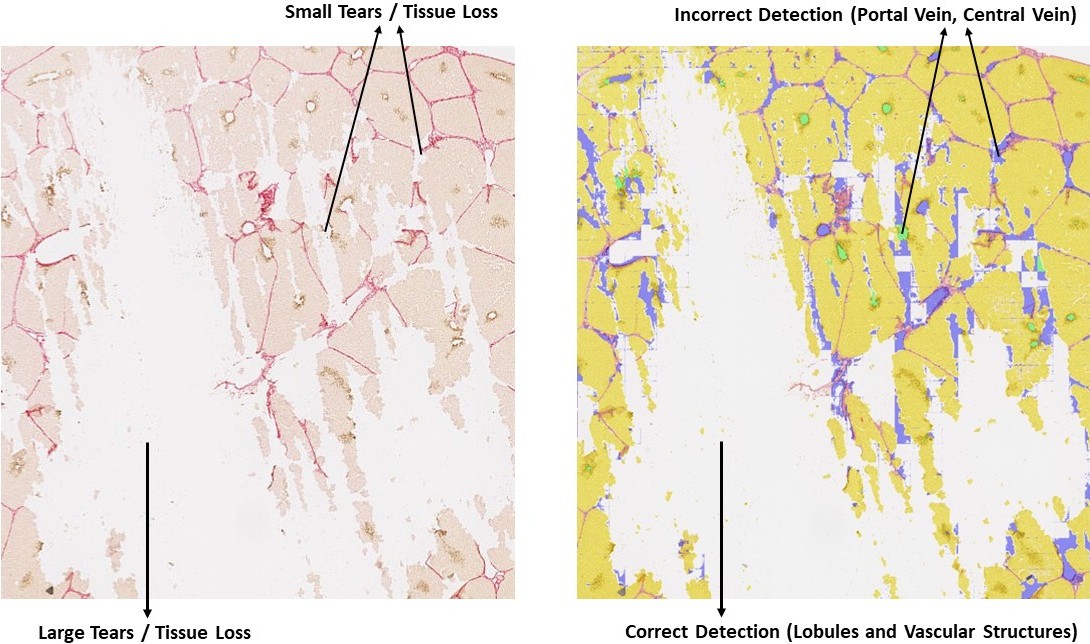

Supplement: Supplementary file 1 [file Image9.jpeg]

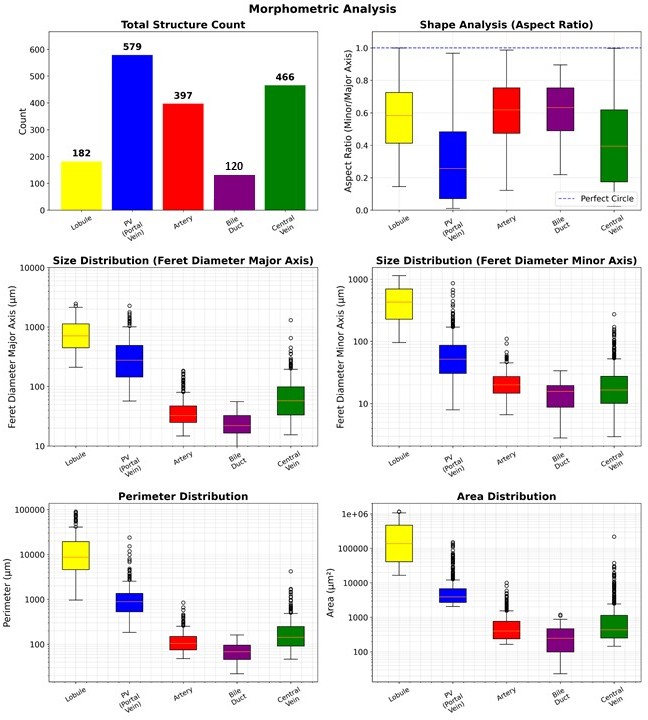

Supplement: Supplementary file 2 [file Image1.jpeg]

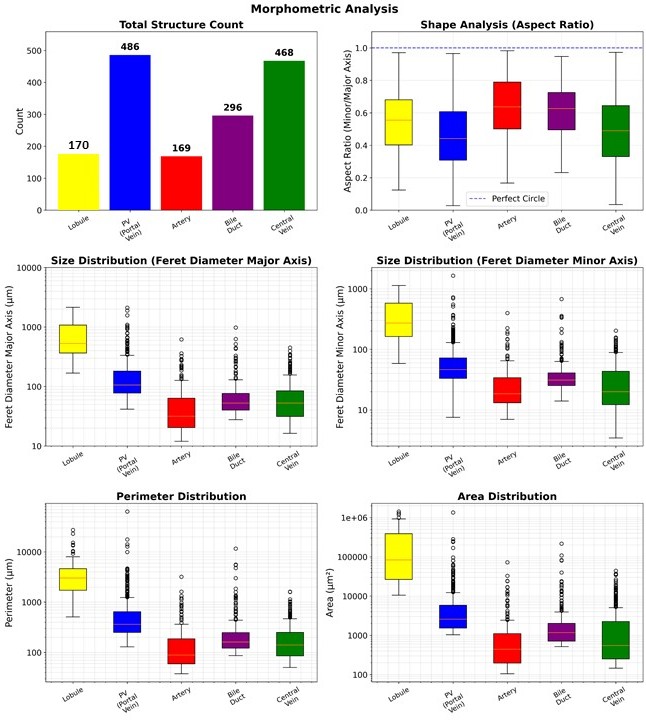

Supplement: Supplementary file 3 [file Image4.jpeg]

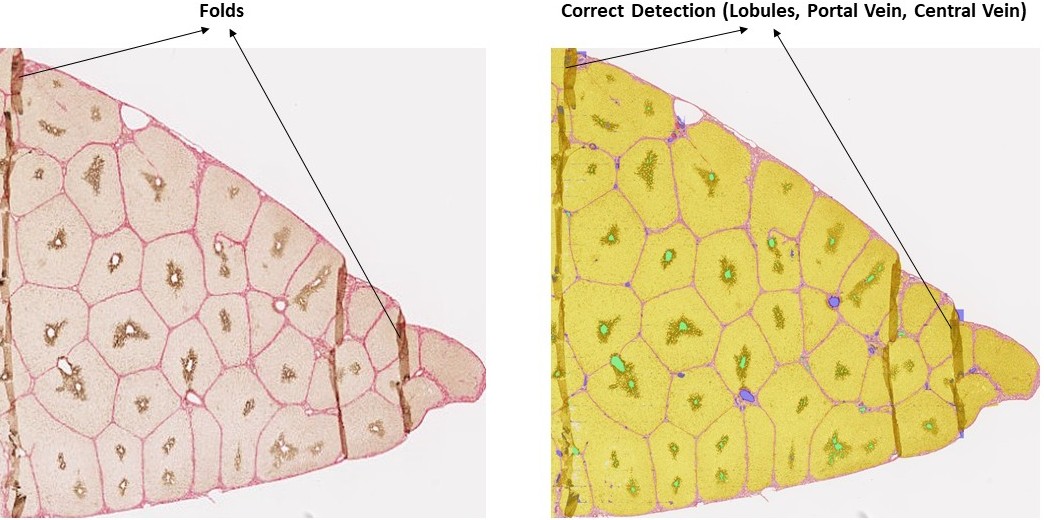

Supplement: Supplementary file 4 [file Image10.jpeg]

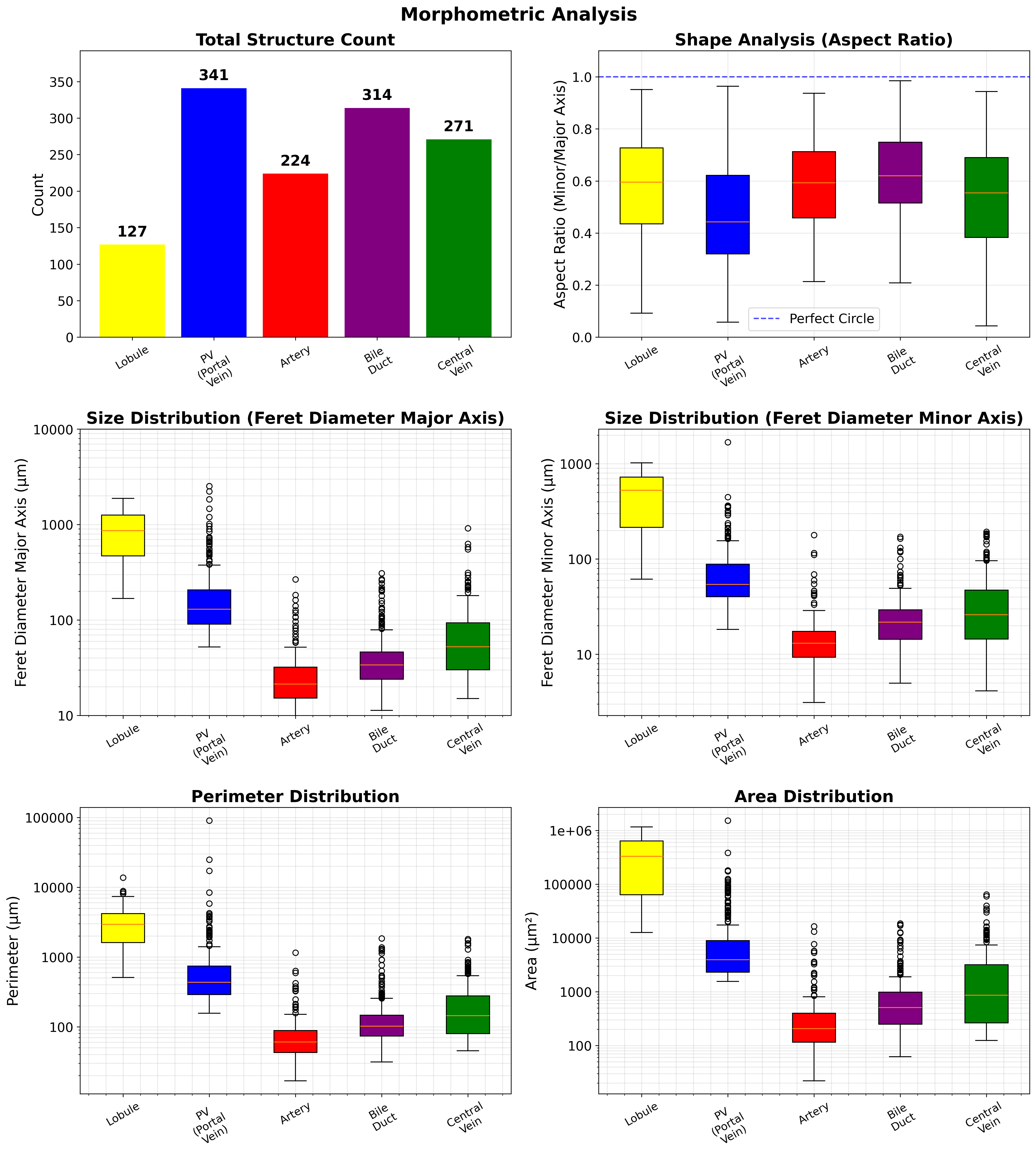

Supplement: Supplementary file 5 [file Image5.png]

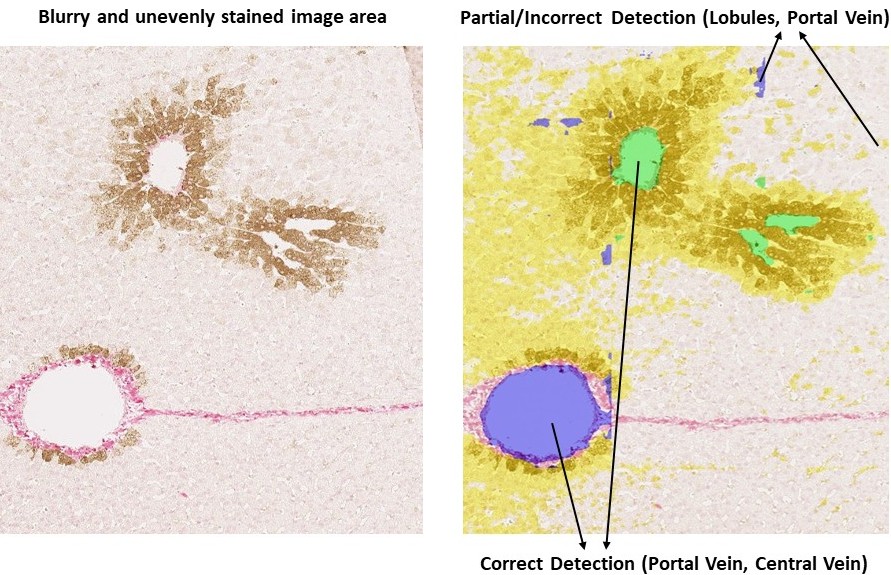

Supplement: Supplementary file 6 [file Image11.jpeg]

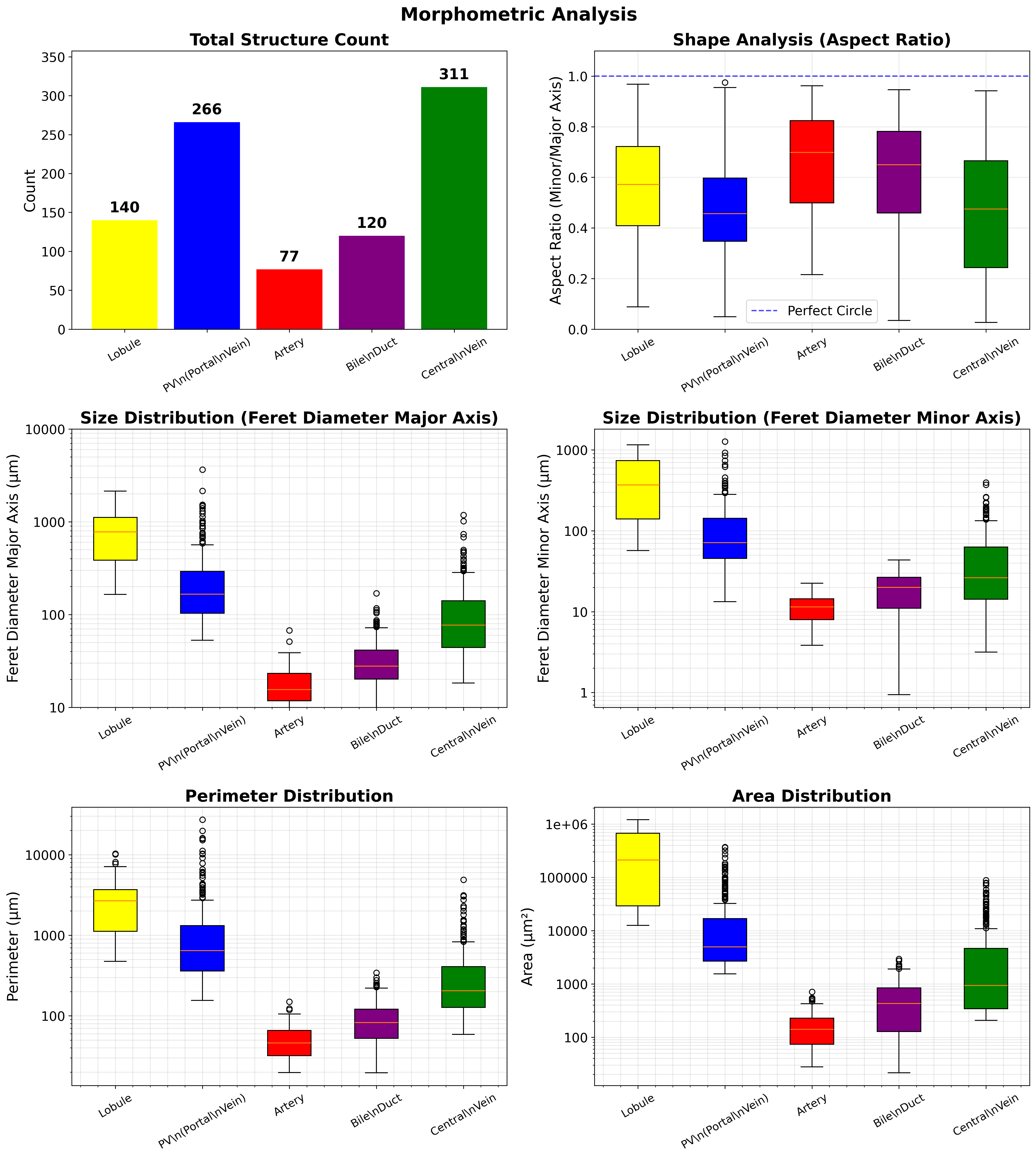

Supplement: Supplementary file 7 [file Image7.png]

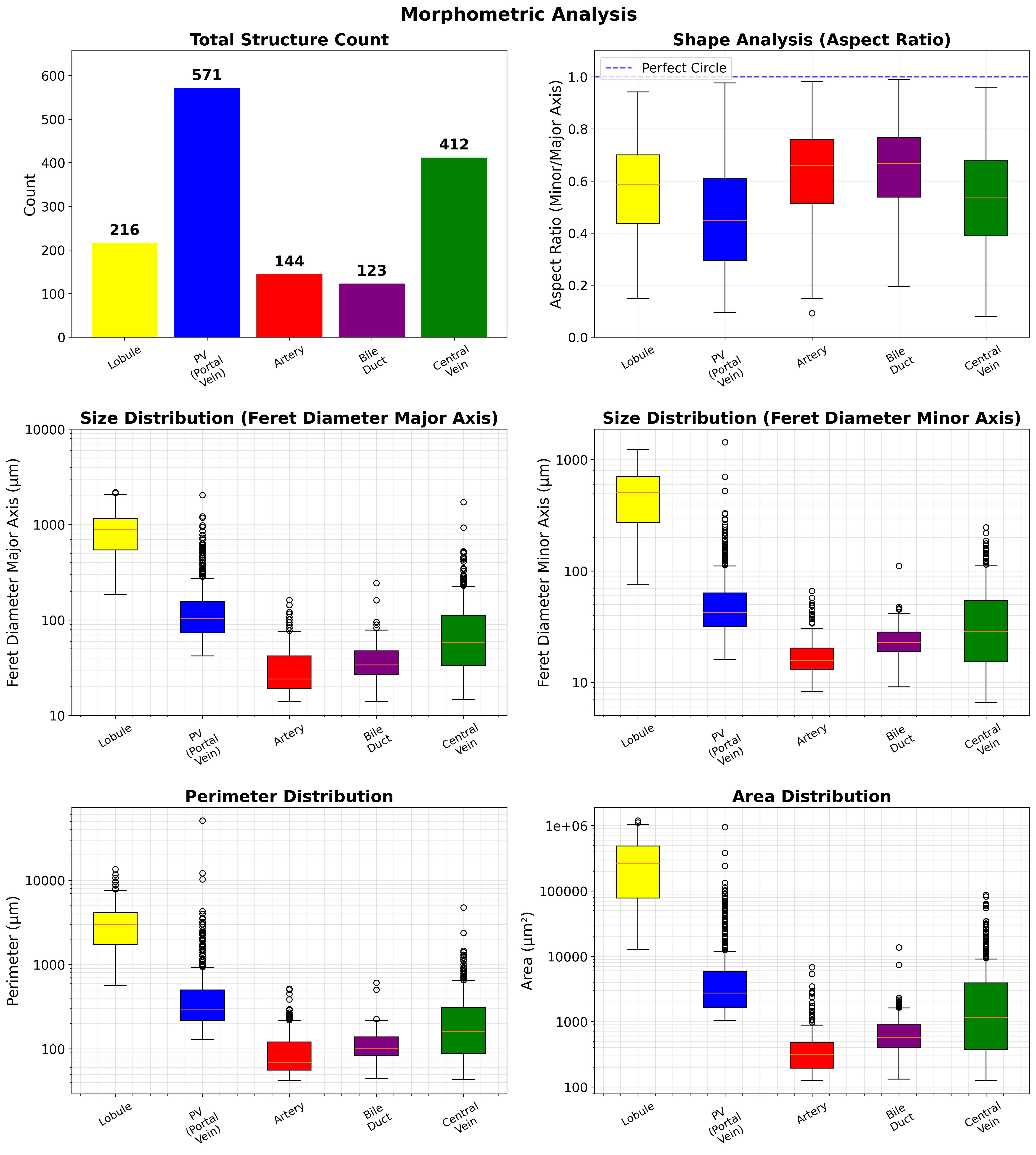

Supplement: Supplementary file 8 [file Image2.png]

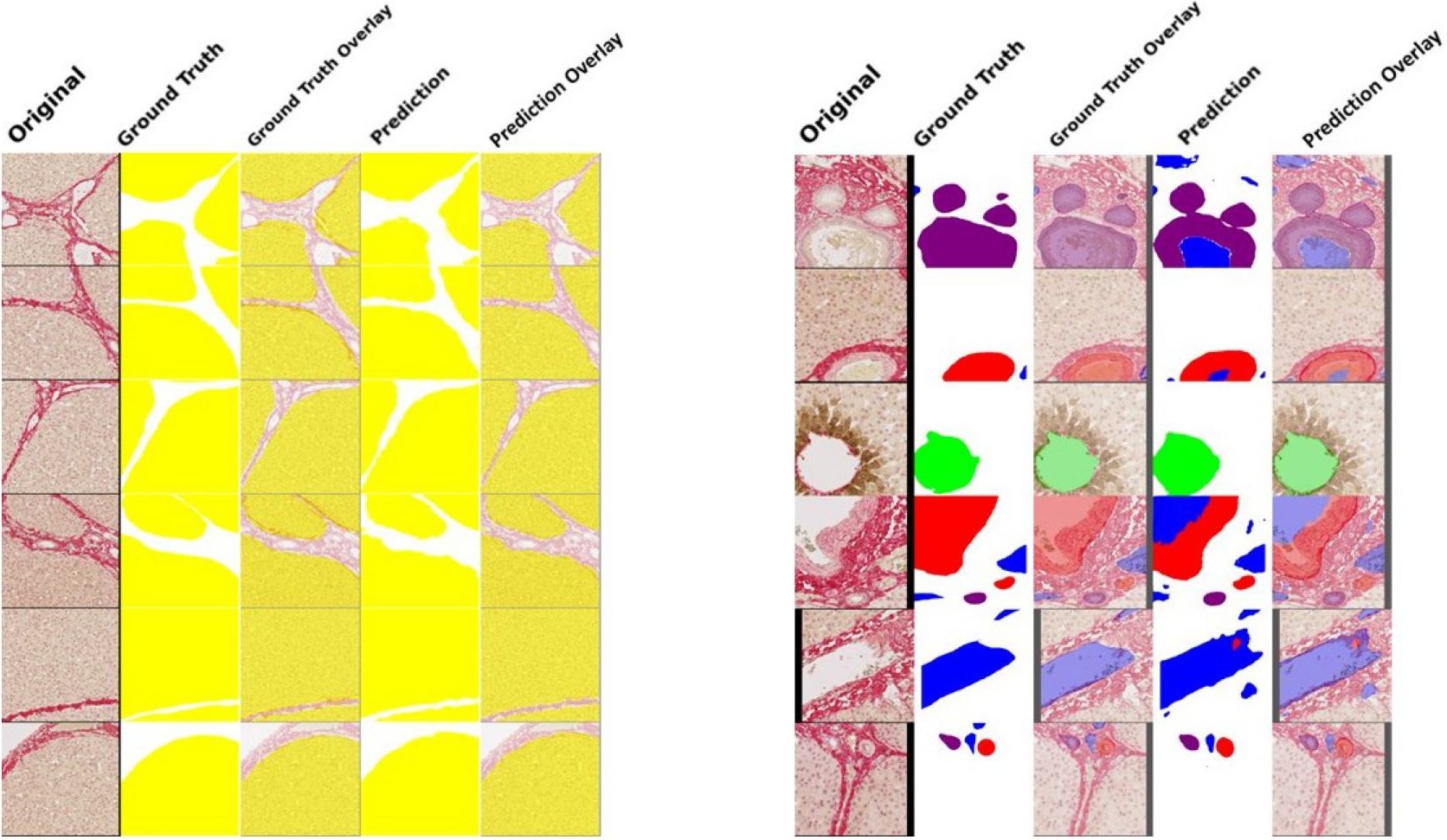

Supplement: Supplementary file 9 [file Image8.jpeg]

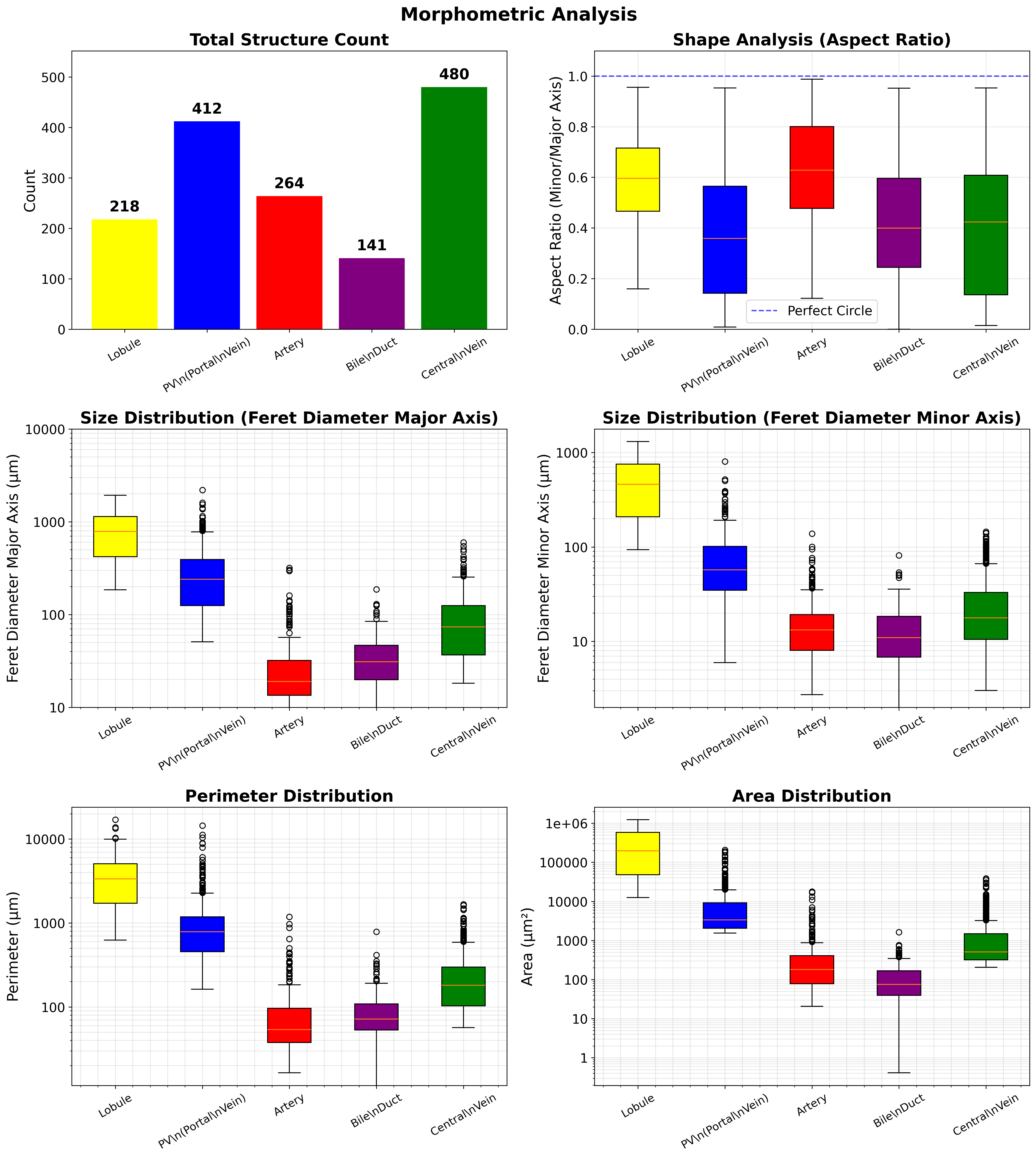

Supplement: Supplementary file 10 [file Image6.png]

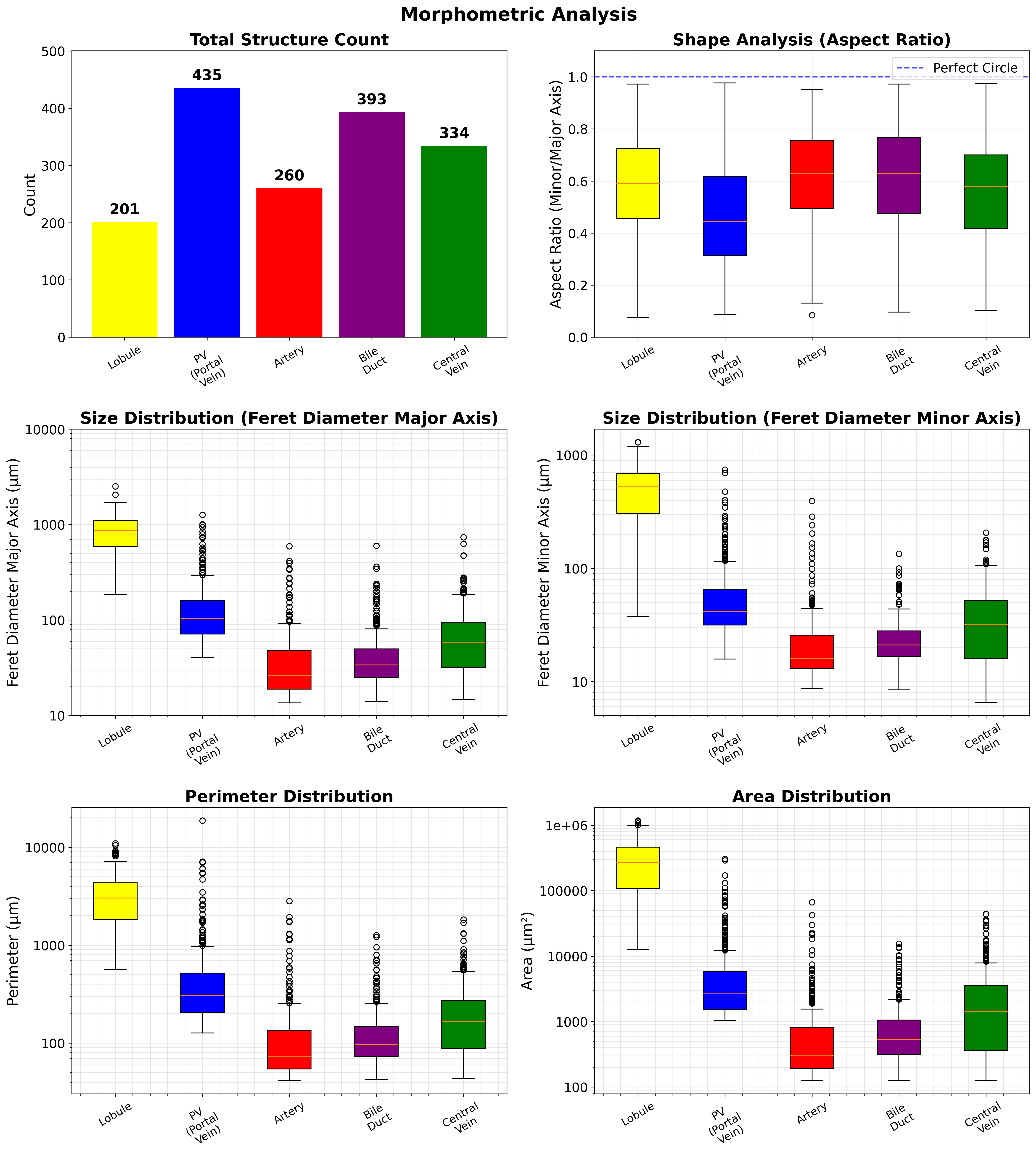

Supplement: Supplementary file 11 [file Image3.png]
